# Supplementary material for: The involvement of attentional biases in endogenous pain inhibition and autonomic reactivity
Source: PLoS One. 2026 Feb 23;21(2):e0342113. doi: 10.1371/journal.pone.0342113 (PMC12928395; doi:10.1371/journal.pone.0342113)
Supplement: S1 Text — Detailed description of the attentional bias paradigms. (PDF) [file pone.0342113.s001.pdf]

Supplementary Materials

Detailed description of the attentional bias paradigms.

1. Modified Perceptual Load task. Participants performed a pain modification of the Perceptual Load paradigm (Lavie, 1995), based on previous studies conducted in our laboratory (Okon-Singer et al., 2007, 2011). The experiment was created using E-Prime 3.0 software.

Participants indicated whether a target letter (X or N) appeared on the screen by responding to the corresponding letter on the keyboard. The task contained two perceptual load conditions, low and high, which were determined by the number of distracting letters appearing alongside the target letter. In the low load condition, only the target letter appeared on the screen, whereas in the high load condition, four distracting letters appeared on the screen together with the target letter. The distracting letters were chosen randomly from the letters K, H, V, Z, or W. The target and distracting letters appeared randomly in six possible locations, creating an imaginary circle at the center of the screen.

Each trial started with a fixation cross that appeared for 500 milliseconds (ms). Then the letters appeared on the screen for 1,350 ms or until a response. Simultaneously with the letters' onset, a distracting picture was presented in the middle of the screen for 200 ms. The picture was either neutral or pain-related (e.g., a hand being cut by a knife, a person holding their stomach with a painful expression, or a visible sports injury). Participants were instructed to ignore the picture and indicate if and which target letter appeared, as quickly and as accurately as possible, using the keyboard. To maintain participant alertness, 10% of the trials were "catch trials" with no target letter. In these trials, participants were instructed not to respond.

Accuracy percentages and reaction times (RTs) were recorded from the onset of the target to the keypress. Pain-related interference bias in the task was calculated by subtracting the mean RT for trials with neutral interfering images from those with pain-interfering images, separately for the low and the high conditions, creating two separate interference bias indexes (i.e., Pain interference, Low Perceptual Load, and Pain interference, High Perceptual Load).

Picture selection for the Perceptual Load task. The distracting pictures were specifically selected for this experiment and evaluated by a separate cohort of participants. The pictures were taken from freely available pictures on the web. All pictures were resized to 254 X 338 pixels and were edited to a circle shape using Patin.net 5.0.9 software. Both picture types were matched for luminance and contrast. Prior to the main study, both picture valences (i.e. pain-related and neutral) were rated by twenty unrelated participants, who were required to rate each picture for pain intensity levels, unpleasantness, and disgust, on a VAS scale ranging from 1 (not painful at all / no unpleasantness / no disgust), to 7 (maximum pain / highly unpleasantness / most disgusting). For the pain-related pictures, only pictures rated as more painful than 2.5 on the VAS pain scale and got higher pain ratings than disgust ratings were selected for the experiment. For the neutral pictures, only pictures with an average of pain, unpleasantness, and disgust of less than 1.5 were selected to be included. A t-test analysis verified that all pain-related pictures were rated as more painful [Mean pain intensity pain-related pictures = 3.83; Mean pain intensity neutral pictures = 1.02; $t_{(79)}$=19.89, p<.001] and unpleasant than neutral pictures [Mean unpleasantness pain-related pictures = 3.83; Mean unpleasantness neutral pictures = 1.02; $t_{(79)}$=15, p<.001].

2. Dot-Probe task. We employed a modified version of the Dot-probe task previously used in pain research (Schoth et al., 2013; Sharpe et al., 2013) to assess attention bias toward pain-related words. The task measures attention bias by simultaneously displaying one pain word

and one neutral word on the screen, after which one of them is replaced by a letter probe. In this task, although participants were not required to respond to pain or neutral stimuli directly, attentional capture is inferred from RT to the probe appearing in a location previously occupied by either a pain or a neutral word.

The task was programmed using PsychoPy. Each trial began with a 500 ms fixation cross, and participants were instructed to maintain their gaze on this location throughout the task. A pair of words, one pain and one neutral, was then presented above and below fixation for 500 ms. The letter height was 29.9 cm, and the centers of the words were positioned 2.24 cm above and below the center of the screen, respectively. After the words disappeared, a probe letter ('p' or 'q') appeared in the location of one of the words. Participants were asked to identify the probe letter as quickly and accurately as possible by pressing the corresponding key on the keyboard, and accuracy rates and RTs were recorded. The probe disappeared after a response or after 1 second, followed by a 500 ms inter-trial interval.

The probe appeared equally often in the locations of pain-related and neutral words. Word and probe positions (top or bottom) were counterbalanced within participants, yielding four trial types: pain-related word top/probe top, pain-related word top/probe bottom, pain-related word bottom/probe bottom, and pain-related word bottom/probe top. Assignment of words and probe positions was randomized across trials. Pain-congruent trials were defined as trials in which probes replaced the pain-related words; pain-incongruent trials were defined as trials in which probes replaced neutral words. To reduce familiarity effects, each word pair was shown only three times. The task included 10 practice trials using neutral-pain pairs not used in the test phase, followed by 90 experimental trials.

Dot-probe attention bias was calculated only on trials with accurate responses, by subtracting the average RT for pain-congruent trials (probe in the location of the pain word) from pain-incongruent trials (probe in the location of the neutral word).

Words for the dot-probe task. *A* set of word stimuli was specifically created, consisting of 34 pain/neutral word pairs. These pairs were matched for length and frequency in Hebrew using a word frequency corpus developed by the Israeli National Institute for Testing and Evaluation (NITE). This approach aligns with previous studies employing pain-related words in dot-probe tasks (Dehghani et al., 2003; McGowan et al., 2009).

The pain-related words were selected from prior cognitive tasks assessing attention bias to pain (e.g., dot-probe; Bowler et al., 2017; Dehghani et al., 2003; McGowan et al., 2009; Sharpe et al., 2012; Van Ryckeghem et al., 2018), Stroop tasks (Andersson & Haldrup, 2003; Asmundson et al., 2005)) and pain questionnaires (e.g., the McGill pain questionnaire; Melzack, 1987) and were translated into Hebrew. Due to the limited number of distinct Hebrew words representing pain, the 34-word pairs were selected from all four pain-related categories: sensory (e.g., drilling), affective (e.g., agonizing), threat (e.g., torture), and disability (e.g., debilitating). For the complete word list presented in the task, see Table S1.
