# Supplementary material for: The involvement of attentional biases in endogenous pain inhibition and autonomic reactivity
Source: PLoS One. 2026 Feb 23;21(2):e0342113. doi: 10.1371/journal.pone.0342113 (PMC12928395; doi:10.1371/journal.pone.0342113)
Supplement: S1 Table — (PDF) [file pone.0342113.s002.pdf]

S1 Table. List of words used in the dot probe task.

Hebrew pain word (English)	Neutral word (English)
(Agonizing) מייסר	(Binding) כריכה
(Vulnerable) פגיע	(Fortress) מבצר
(Disease) מחלה	(Conversation) שיחה
(Paralysed) משותק	(dressed) מתלבש
(Ill) חולה	(Star) כוכב
(Chronic) כרוני	(Transport) הובלה
(Injury) פציעה	(Booklet) חוברת
(Sore) דלקת	(Harvest) קטיף
(Shooting) ירי	(Forest) יער
(Stings) עקיצות	(Openness) פתיחות
(Pounding) חבטות	(Display) תצוגה
(Sharp) חד	(Garden) גן
(Burning) שורף	(Government) ממשל
(Aching) כואב	(Classroom) כיתה
(Beating) מרביץ	(Bonus) בונוס
(Pinching) צביטה	(Nobility) אצולה
(Wound) פצע	(Bear) דוב
(Lethal) קטלני	(Quote) ציטוט
(Killing) הורג	(Food) מאכל
(Suffocating) נחנק	(Mattress) מזרן
(Surgery) ניתוח	(Familiar) להכיר
(Biting) נשיכה	(Anemones) כלניות
(Cutting) חיתוך	(Freedom) חירות
(Itching) עקצוץ	(Token) אסימון
(Stabbing) דקירה	(Clothing) הלבשה
(Stinging) עוקץ	(Spy) מרגל
(Torture) עינוי	(Kiddush) קידוש
(Bruised) פצוע	(Pillow) כרית
(Agony) יסורים	(conductor) כרטיסן
(Spasm) התקף	(Wheel) גלגל
(Ache) כאב	(Luck) מזל
(Suffer) לסבול	(Objects) חפצים
(Cut) חתך	(Tail) זנב
(Emergency) חירום	(Meeting) פגישה
(Attack) תקיפה	(Farmer) חקלאי
(Hazard) מפגע	(Bathtub) אמבט
(Casualty) נפגע	(Painting) ציור
(Cruel) אכזרי	(Heritage) מורשת
(Debilitating) מגביל	(Praises) שבחים
(Penetrating) חודר	(Tennis) טניס
(Throbbing) פועם	(Running) ריצה
(Drilling) קודח	(Box) תיבה
(Hurting) פוגע	(Temple) מקדש
(Crushing) מרסק	(Flowerpot) עציץ
(Damage) נזק	(Village) כפר

(Hazardous) מסוכן	(Sport) ספורט
(Harmful) מזיק	(Messenger) שליח
(Itching) מגרד	(Holidays) חגים
(Pulling) מורט	(Godfather) סנדק
(Tingling) מעקצץ	(Umbrella) מטריה
(Grinding) שוחק	(Piyyut) פיוט
(Stabbing) דוקר	(Hair) שיער
(Intense) עוצמתי	(Quotation) מרכאות
(Painful) מכאיב	(Vote) הצבעה
(Pinching) צובט	(Jugs) כדים
(Squeezing) מועך	(Buckle) אבזם
(Biting) נושך	(Producer) מפיק
(Troublesome) טורדני	(read) להקריא
(Injured) פוצע	(Trend) טרנד
(Interfere) מפריע	(Neck) צוואר

Note. Hebrew pain-related and neutral words used in the study are presented in the table below. For clarity, English translations are provided in parentheses directly next to each Hebrew word.
