# Supplementary material for: The involvement of attentional biases in endogenous pain inhibition and autonomic reactivity
Source: PLoS One. 2026 Feb 23;21(2):e0342113. doi: 10.1371/journal.pone.0342113 (PMC12928395; doi:10.1371/journal.pone.0342113)
Supplement: S2 Table — (PDF) [file pone.0342113.s003.pdf]

S2 Table. Mean accuracy and reaction time performance in the attentional bias tasks.

	**Mean Accuracy, % (*SD, 95% CI*)**	**Mean Reaction time, ms (*SD, 95% CI*)**
**Pain interference - Low Perceptual Load**	Pain interference images: 0.93, (*0.05; 95% CI, 0.92 to 0.95*)<br>Neutral interference images: 0.94, (*0.04; 95% CI, 0.93 to 0.95*)<br>Overall in both conditions: 0.94 (*0.04; 95% CI, 0.93 to 0.95*)	Pain interference images: 541.3 (*56.3; 95% CI, 527.0 to 550.9*)<br>Neutral interference images: 532.2 (*57.1; 95% CI, 517.8 to 541.2*)<br>Overall, in both conditions: 536.7 (*56.6; 95% CI, 524.8 to 548.7*)
**Pain interference - High Perceptual Load**	Pain interference images: 0.84 (*0.08; 95% CI, 0.83 to 0.86*)<br>Neutral interference images: 0.82 (*0.10; 95% CI, 0.80 to 0.85*)<br>Overall in both conditions: 0.83 (*0.08; 95% CI, 0.81 to 0.85*)	Pain interference images: 933.6 (*87.5; 95% CI, 911.9 to 949.7*)<br>Neutral interference images: 925.0, (*89.1; 95% CI, 904.0 to 942.4*)<br>Overall in both conditions: 929.4 (*88.2; 95% CI, 911.0 to 947.7)*
**Dot-probe attention bias**	Pain-congruent trials: 0.96 (*0.05; 95% CI, 0.95 to 0.97*)<br>Pain-incongruent trials: 0.96 (*0.04; 95% CI, 0.95 to 0.97*)<br>Overall in both conditions: 0.96 (*0.04; 95% CI, 0.95 to 0.97*)	Pain-congruent trials: 493.8 (*64.6; 95% CI, 478.3 to 503.5*)<br>Pain-incongruent trials: 494.5 (*61.44; 95% CI, 476.8 to 503.9*)<br>Overall in both conditions: 494.1 (*62.0; 95% CI, 480.6 to 507.7*)
