# Supplementary material for: The involvement of attentional biases in endogenous pain inhibition and autonomic reactivity
Source: PLoS One. 2026 Feb 23;21(2):e0342113. doi: 10.1371/journal.pone.0342113 (PMC12928395; doi:10.1371/journal.pone.0342113)
Supplement: S3 Table — (PDF) [file pone.0342113.s004.pdf]

S3 Table. Correlations between attentional bias indexes, psychological traits, and emotional state.

	Dot-probe attention bias	Pain interference - Low Perceptual Load	Pain interference - High Perceptual Load	FPQ-9[a]	PCS[b]	DASS[c]	Baseline HR[d]	%HR change during CPM reactivity[e]
**Dot-probe attention bias**	-	-0.16	0.11	-0.27*	0.01	-0.13	0.05	0.29*
**Pain interference - Low Perceptual Load**	-0.16	-	0.01	0.19	0.10	0.06	-0.03	-0.16
**Pain interference - High Perceptual Load**	0.11	0.01	-	0.06	0.01	-0.03	-0.06	-.02
**FPQ-9[a]**	-0.27*	0.19	0.06	-	.41**	0.24	-0.02	-.20
**PCS[b]**	0.01	0.10	0.01	.41*	-	0.48*	0.05	-0.19
**DASS[c]**	-0.13	0.06	-0.03	0.24	0.48*	-	0.04	-0.35*
**Baseline HR[d]**	0.05	-0.03	-0.06	-0.02	0.05	0.04	-	-0.28*
**%HR change during CPM reactivity[e]**	0.29*	-0.16	-.02	-.20	-0.19	-0.35*	-0.28*	-

[a] FPQ-9 = Fear of Pain Questionnaire – 9-item version.

[b] PCS = Pain Catastrophizing Scale

[c] DASS = Depression, Anxiety, and Stress Scale – Short Form.

[d] Baseline HR = heart rate during 5 minutes rest at the beginning of the experiment.

[e] *%HR change during CPM reactivity = the difference between HR during CPM reactivity and HR at baseline before CPM, divided by the baseline HR.*

* *p<.05;* ** *p<.01;* *** *p<.001, Bonferroni corrected p-values.*
