# Supplementary material for: The involvement of attentional biases in endogenous pain inhibition and autonomic reactivity
Source: PLoS One. 2026 Feb 23;21(2):e0342113. doi: 10.1371/journal.pone.0342113 (PMC12928395; doi:10.1371/journal.pone.0342113)
Supplement: Attachment — Submitted filename: Summary.docx [file pone.0342113.s005.docx]

**Summary**

This study (N=86 healthy women) tests whether attentional biases (Perceptual Load images, Dot-Probe words), psychological traits (DASS-21, PCS, FPQ), and autonomic activity (heart rate; baseline/reactivity/recovery) explain individual differences in pain sensitivity and conditioned pain modulation (CPM). Key findings: stronger interference by pain cues under high load → higher pain sensitivity; Dot-Probe avoidance → higher fear of pain; none of the examined factors predicted CPM magnitude. HR increased during CPM and stayed elevated in recovery; higher distress related to blunted HR reactivity; attentional indices predicted higher HR during recovery.

**Strengths**

- **Clear gap and two-task design.** Nicely motivates moving beyond attentional *focus* to *biases*, using complementary tasks that target earlier capture vs strategic avoidance.
- **Well-specified QST/CPM and ECG stages.** Baseline/reactivity/recovery windows and repeated-measures analyses are clear; HR effects are quantified.
- **Integration of psychology and ANS.** Parallel treatment of distress, fear, catastrophizing with HR dynamics strengthens interpretation.

**Areas for Improvement:**

1. The fact that attentional biases wasn’t correlated with CPM is unexpected. I would suggest authors to make a more scholarly discussion of this. Which could include the fact that task/modality/time differences could dilute shared variance; attentional tendencies may relate more to autonomic recovery than to CPM magnitude, which is consistent with your recovery HR findings; and most importantly that restricted range in healthy young women reduces covariance.
2. The authors have done an exemplary job providing comprehensive methodological details, which greatly enhances transparency and reproducibility. However, the level of detail in the main text occasionally impedes narrative flow and may overwhelm readers unfamiliar with attentional bias paradigms or QST protocols. I recommend moving some procedural specifics to supplementary materials while retaining core conceptual information in the main text. For example, Section 2.3.1.a (Perceptual Load task, lines 156-182) could be streamlined to emphasize *what* the task measures (early attentional capture by pain stimuli under varying cognitive load) and *why* it was chosen, with stimulus presentation parameters, letter selection criteria, and catch trial percentages moved to a supplement. The same could be done for other procedures. This restructuring would help readers grasp the conceptual logic—how attentional biases were operationalized and measured—without getting lost in technical specifications, while still providing the detail necessary for replication.
3. While the authors report η² for their ANOVA analyses, I recommend also including Cohen's d effect sizes for key pairwise comparisons and t-tests to facilitate meta-analytic integration and practical interpretation. It is recommended to report both η² for omnibus tests and Cohen's d (or Hedges' g for small samples) for specific contrasts, as this provides readers with both variance explained and the magnitude of differences in standard deviation units. This is particularly important given that some findings (e.g., attentional biases predicting pain sensitivity) represent novel contributions that future researchers will want to interpret and replicate.
4. The sentence “Specifically, the ANS is involved in shaping CPM efficacy, with studies showing increased 96 heart rate (HR) and sympathetic activation during CPM: should be revised to distinguish group-level HR increases “during CPM” from between-person prediction. Do authors mean that the expected within-task HR rise or that individual HR reactivity predicts CPM magnitude.
5. At first mention in Participants, add a parenthetical brief explanation regarding SONA, clarifying its meaning. This makes recruitment transparent for international readers and non-academic audiences.
6. Authors describe that normality was checked using Shapiro-Wilk and histograms but don’t describe what was done if violated.

**Minor Comments:**

1. The resolution (DPI) of the figures appears to be below publication standards. Panel labels in Figure 6 (A, B, C) are difficult to read, and the violin plot contours show pixelation.
2. The OSF data repository link provided (<https://osf.io/3bwy7/files/osfstorage>) does not appear to be functioning properly or may not be publicly accessible. When accessing the link, it does not resolve to a viewable repository page. I believe the correct link should be: <https://osf.io/3bwy7/files> or <https://osf.io/3bwy7>.

This manuscript represents a methodologically rigorous and theoretically well-motivated investigation into the role of attentional biases in pain sensitivity and endogenous pain modulation. The study makes meaningful contributions by demonstrating that attentional biases predict pain sensitivity independently of psychological factors, and by revealing their association with autonomic recovery dynamics, novel findings that advance our understanding of cognitive mechanisms in pain processing. The use of complementary attentional bias paradigms, careful QST/CPM methodology, and integration of autonomic measures are particular strengths. The suggested revisions focus primarily on enhancing clarity in the Discussion of unexpected null results, improving readability through strategic reorganization of methodological detail, strengthening effect size reporting, and addressing minor technical issues with figures and data accessibility.
