# Supplementary material for: The involvement of attentional biases in endogenous pain inhibition and autonomic reactivity
Source: PLoS One. 2026 Feb 23;21(2):e0342113. doi: 10.1371/journal.pone.0342113 (PMC12928395; doi:10.1371/journal.pone.0342113)
Supplement: Attachment — Submitted filename: Response to Reviewers.docx [file pone.0342113.s007.docx]

**Response letter to the reviewers’ comments**

Manuscript ID: PONE-D-25-45053

The involvement of attentional biases in endogenous pain inhibition and autonomic reactivity

Dear Prof. Xianwei Che, Academic Editor, and Reviewers,

We sincerely thank the reviewers for their thoughtful, constructive, and highly encouraging feedback on our manuscript. We truly appreciate the kind words and the evident enthusiasm and engagement shown in reading our work. The comments provided were insightful and have significantly strengthened the quality and clarity of the manuscript.

We have addressed all concerns raised by the reviewers in a point-by-point manner below. All revisions are marked using highlights in the updated manuscript (and cited within the corresponding responses for clarity).

**Reviewer #1**

*“The authors investigated the interplay between pain-related attentional biases, psychological traits, heart rate as a measure of autonomic reactivity and conditioned pain modulation (CPM). The relevance of the current study becomes clear in the context of previous research; the authors did a great job in summarizing the state of the art and identifying important gaps in knowledge which they attempted to close.*

*From my perspective, this study has several important strengths that I would like to point out before sharing my criticism:*

- *The authors used two different paradigms for assessing attentional bias combined with two different sets of stimuli (pain-related words vs. pictures). This approach is warranted by previous research and led to the identification of a very interesting pattern in results, which would have been missed when using just one of the two paradigms.*
- *The inclusion of heart rate in their analyses yielded an innovative result linking attentional bias and psychological distress to autonomic reactivity under CPM/painful stimulation which is highly relevant and stimulates further investigation of these associations.*
- *The overall writing style is clear and concise, the theoretical and empirical background is presented extensively, and the statistical analyses are thorough and suitable for testing the authors' hypotheses.*”

**Comment 1:** “*When the authors state their hypothesis at the end of the Introduction, the hierarchy of and potential interaction between the different predictors does not become entirely clear (‘We hypothesized that individuals with stronger attentional biases toward pain, higher levels of pain-related distress, and heightened autonomic reactivity during CPM would exhibit reduced endogenous pain inhibition’). In contrast, in the first paragraph of the Discussion it is clearly stated that attentional bias is treated as the main predictor, and it was explored whether the other factors provide ‘additional explanatory value’. I would recommend adjusting the wording in the Introduction accordingly to make this clearer. This might also apply to the Abstract.”*

**Response:** We thank the reviewer for this important and constructive comment. We agree that clarifying the hierarchy of predictors in the introduction and abstract will improve the manuscript. Accordingly, we have revised the wording of the study aims and hypothesis to clearly indicate that attentional bias is treated as the primary predictor, while psychological, emotional, and autonomic factors are examined for their additional explanatory value. These revisions now align more closely with the summary lines presented in the discussion.

**Introduction, Page 6, paragraph 3:** ”This study aimed to determine whether attentional bias accounts for individual differences in pain sensitivity and endogenous pain modulation, and to what extent psychological, emotional, and autonomic factors offer additional explanatory power. We focused particularly on CPM as an index of endogenous pain inhibition and HR dynamics as a marker of autonomic activity. We primarily hypothesized that individuals with stronger attentional biases toward pain would exhibit reduced endogenous pain inhibition. In addition, we expected that higher levels of pain-related distress, heightened autonomic reactivity during CPM, would contribute secondary, complementary effects, further explaining individual variability in endogenous pain modulation.”

**Abstract, Page 2:** “… The present study examined whether attentional biases predict individual differences in pain sensitivity and endogenous pain modulation, and whether psychological, emotional, and autonomic factors are associated with these outcomes.”

**Comment 2*:*** *“I was confused by the following sentence: ‘Specifically, the ANS is involved in shaping CPM efficacy, with studies showing increased heart rate (HR) and sympathetic activation during CPM’ (Introduction, p. 5) as the first part of the sentence does not logically follow from the second part. Did these studies only show that heart rate was elevated during CPM, or are there clear indicators for an involvement in CPM efficacy (e.g., participants with higher heart rate showing more efficient CPM)? It would be important to clarify this.”*

**Response:** We thank the reviewer for highlighting this ambiguity. We revised the section to clearly distinguish evidence for autonomic nervous system (ANS) predictors of CPM efficacy from autonomic reactivity during the CPM task itself. The revised text now notes that several studies have linked resting vagal tone and sympathetic reactivity during test pain alone to CPM magnitude, supporting a predictive role of ANS activity in CPM efficacy. In contrast, only a small number of studies have assessed autonomic changes during the CPM paradigm, reporting increased sympathetic activation and reduced vagal tone at the group level. Importantly, only one of these studies examined the association between autonomic reactivity during CPM and CPM magnitude and found no significant relationship. This revision resolves the inconsistency and clarifies the distinction between predictive and within-task autonomic indicators.

**Page 5, Paragraph 3:** “In addition, autonomic nervous system (ANS) activity is increasingly recognized as a key contributor to the subjective experience of pain [19,99] and pain inhibition [13,53,63]. Specifically, initial evidence suggests that the ANS may be involved in shaping CPM efficacy. A few studies have linked resting vagal tone, measured via heart rate variability (HRV) or blood pressure, to later CPM magnitude, with higher vagal tone predicting a larger CPM effect [69,72,76]. Other work highlights the role of sympathetic activation, showing that greater sympathetic reactivity during pain alone is associated with enhanced CPM efficacy [15,119]. At the same time, cardiovascular autonomic dynamics during CPM have been investigated in only two studies to date [5,51]. Both reported group-level decreases in vagal tone and increases in sympathetic activation during the CPM procedure; however, only one study examined whether such changes were associated with CPM magnitude, and it found no significant correlation [51]. “

**Comment 3.a:** *“My main concerns relate to the paradigm used for CPM/painful stimulation. Here, I noticed a few aspects that are (from my experience) unusual and might be problematic:
a) Individually adjusting the intensity of the test stimuli to a level of 60 seems reasonable; however, I am not sure about the reliability of the authors’ methods. In my experience, it takes quite some time to familiarize participants with the thermode and several practice runs so they learn to differentiate between non-painful and painful sensation and to reliably use the rating scale. Applying three single stimuli to determine the approximate location of a ‘60’ on the rating scale (then followed by three more) seems insufficient. Unfortunately, the ratings of the test stimuli (Table S 2 in the Supplementary Material) underscore this problem, with ratings decreasing dramatically from 60 to 20-25 within the 60 seconds of stimulation. In my eyes, this suggests that the initial rating might be exaggerated by fear/startle. The authors should at least discuss this and the descriptive statistics of the test stimulus ratings should be presented in the Results section (not in the Supplementary Material) as they are the main outcome measure of this study”.*

**Response:** The reviewer raises an important methodological consideration, highlighting the need to clarify our procedure for determining the pain-60 stimulus. We agree that the original description may have given the impression that only a limited number of trials were used, which could raise concerns regarding reliability and participant familiarization.

In practice, the calibration procedure was lengthy and iterative, typically lasting approximately 15 minutes. Participants were exposed to repeated blocks of three heat stimuli at varying temperatures, and temperature adjustments were made between blocks based on the participants’ numerical pain scale (NPS) ratings, following commonly used procedures (e.g., Granot et al., 2008; <https://doi.org/10.1016/j.pain.2007.06.029>). This process was repeated until the same temperature elicited an NPS rating of approximately 60 in three separate repetitions, at which point it was accepted as the participant’s pain-60 level. This approach ensured adequate familiarization with both the thermode and the rating scale, as well as stability of the selected stimulus intensity. We have revised the Methods section to describe this procedure more explicitly.

It should also be noted that although a fixed stimulus intensity is determined based on pain-60—reflecting the perceived pain intensity at the onset of stimulation—pain ratings during prolonged tonic heat stimulation are expected to vary over time as a function of stimulus duration and stimulation sequence. In our sample, ratings declined on average from the initial target value (~60) toward lower levels (approximately 20–25) over the 60-second stimulation period, with substantial interindividual variability. Such temporal changes in pain ratings are well documented in prolonged heat pain paradigms and reflect known dynamics of pain perception, including adaptation or sensitization depending on stimulus temperature and individual pain sensitivity (e.g., Weissman-Fogel et al., 2015; <https://doi.org/10.1002/ejp.562>), rather than exaggerated initial ratings due to fear or startle alone. Importantly, higher pain-60 temperatures are typically associated with greater sensitization, whereas lower pain-60 temperatures tend to be associated with adaptation. However, this does not compromise the validity of the CPM measure, as CPM is computed using a subtraction-based index that inherently accounts for baseline changes and adaptation effects.

Furthermore, in response to the reviewer’s suggestion, we have moved the descriptive statistics of the test-stimulus ratings from the Supplementary Material to the Results section, Table 2.

**Page 11, Paragraph 1:** “A 30 × 30 cm Peltier thermode (Thermal Sensory Analyzer-2001, Medoc, Israel) was applied to the volar surface of the dominant forearm, to determine the heat temperature corresponding to pain-60 (i.e., the temperature participants rate as 60 on a 0-100 numerical pain scale (NPS), ranging from '0'=no pain, '100'=worst imaginable pain). Stimulus intensity was individually calibrated such that all participants experienced the same subjective level of pain, as the focus of the study was on pain perception rather than nociceptive input. The pain-60 calibration procedure employed here is consistent with the commonly used protocol in the literature [42]. In general, participants were exposed to a series of hot stimuli of 8-second duration. Participants were first familiarized with the NPS by rating three initial heat stimuli (43 °C, 45 °C, and 47 °C; ramp rate: 2 °C/s from a 32 °C baseline), with a 20-seconds inter-stimulus interval. After each stimulus, participants were asked to report their level of pain on the verbal NPS. Based on these ratings, we identified an initial temperature with the NPS score closest to 60, with which we continued our pain-60 determination procedure.

The pain-60 determination then continued with a series of blocks containing three heat stimuli: the closest temperature to the temperature rated as 60 at the introduction phase, one degree lower, and one degree higher (presented in a random order). After each trial, the thermode was repositioned to prevent sensitization. At the end of each rating block, if none of the three temperatures elicited a rating near 60, another block containing three ratings was conducted, with the heat stimuli temperatures adjusted upward or downward in 1 °C increments, again including the adjacent temperatures above and below the new center point.

Importantly, this procedure was iterative: blocks were repeatedly administered and adjusted as needed until the same temperature consistently produced an NPS rating of approximately 60 in three separate trials. This full calibration procedure typically requires around 15 minutes to be completed. Only after achieving such consistency was the temperature defined as the participant’s pain-60, and it was used for the THP stimulation applied alone (“test-stimulus alone”) and during CPM (“test-stimulus under conditioning stimulus”, see below for further details). It should be noted that although a fixed stimulus intensity is used based on the determination of pain-60, which reflects the perceived pain intensity at the onset of stimulation, reported pain ratings typically vary over the course of the test sessions as a function of several factors, including stimulus duration and stimulation sequence [72,130]. Notably, higher pain-60 temperatures are associated with greater sensitization, whereas lower pain-60 temperatures tend to be associated with adaptation; however, this does not compromise the validity of the CPM measure, as CPM is calculated using a subtraction-based index that inherently accounts for adaptation effects.**”**

**Comment 3.b.:** *“Relating to this, please include the stimulus duration of the stimuli that were used to determine ‘pain 60’, the verbal anchors (if any) of the pain scale, and the resulting stimulation intensities for ‘pain 60”.*

**Response:** Thank you for the clarifying request. The stimulus duration, the verbal anchors of the NPS, and the resulting temperatures used for determining pain-60 are important methodological details and were unintentionally omitted in the initial submission. We have now added this information clearly in the Methods section and have also included the descriptive statistics for the resulting pain-60 temperatures in the Results section, as suggested.

**Page 11, Paragraph 1:** …” to determine the heat temperature corresponding to pain-60 (i.e., the temperature participants rate as 60 on a 0-100 numerical pain scale (NPS), ranging from '0'=no pain, '100'=worst imaginable pain). Participants were first familiarized with the NPS by rating three initial 8-second heat stimuli (43 °C, 45 °C, and 47 °C; ramp rate: 2 °C/s from a 32 °C baseline). “

**Page 20, Paragraph 3:** “The pain-60 temperatures used for the THP test ranged between 41-48°C with a mean temperature of 45.04°C (*1.95*).”

**Comment 3.c.:** *“I am not sure why the authors chose to individually tailor the temperature of the hot water bath (conditioning stimulus) as most studies use a fixed temperature of 46 or 46.4 °C. However, please provide some information on the procedure used. How long was the hand immersed during these trials? Could this have influenced the CPM response by potential adaptation to the conditioning stimulus? In addition, the descriptive ratings of CS ratings should also be provided in the Results section.”*

**Response:** We would like to address the reviewer’s concern regarding the individual tailoring of the conditioning stimuli temperature. At the start of data collection, we observed substantial inter-individual variability in pain responses to the fixed temperature of 45.5 °C: for some participants, this temperature was too intense to maintain hand immersion, whereas for others it produced little to no pain. For this reason, based on Nir et al. (2011; <https://doi.org/10.1016/j.ejpain.2010.10.001>), indicating the CPM effect is observed only when hot water immersion leads to moderate or intense pain, we adopted an individualized calibration procedure to ensure that the conditioning stimulus reliably elicited the necessary moderate to intense pain level across all participants (i.e., 30-70 in the NPS). We have now added this rationale clearly to the Methods section.

As suggested, we also clarified the full calibration procedure, including the duration of hand immersion during the trials and the potential influence of adaptation, and added the descriptive statistics for the conditioning-stimulus pain ratings to the Results section. We do not believe that adaptation to the conditioning stimulus influenced the CPM response, as our data show the opposite pattern: pain ratings during the conditioning stimulus increased over the 2.5-minute immersion period rather than decreased, suggesting that adaptation of the CS did not occur. This is now clearly stated in the manuscript. We appreciate the reviewer drawing attention to this point, and we believe these additions improve the clarity and transparency of the methodology.

**Page 13, Paragraph 1:** ”… Following recommendations from the literature [85,136], and evidence indicating the CPM effect is observed only when hot water immersion leads to moderate or intense pain [85], we adopted an individualized calibration procedure to ensure that the conditioning stimulus reliably elicited the necessary moderate to intense pain level across all participants (i.e., 30-70 in the NPS).

For the hot-water pain calibration, participants first immersed their non-dominant hand in a hot water bath set to 45.5 °C (Heto Cooling Bath CBN 8-30, Denmark). After 20 seconds of immersion, they were asked to rate their pain on a 0–100 NPS. If the rating fell within the target range of 30–70, no further adjustments were made. If the rating exceeded or fell below this range, the water temperature was adjusted by 0.5–1 °C in the appropriate direction, and the procedure was repeated. This iterative calibration continued until the participant reported a pain rating within the desired range. This individualized approach was implemented to minimize floor or ceiling effects in the conditioning stimulus and to ensure comparable subjective pain intensity across participants. In our sample, water temperature ranged between 44-47°C [mean temperature of 45.24°C (*0.74*)]. “

**Page 20, Paragraph 3:** …”The hot-water conditioning stimulus produced an average NPS rating of 53.89 (*SD = 16.02*). Pain ratings collected after the first 20 seconds of hand immersion averaged 44.55 (*SD = 16.57*) and increased over the 2.5-minute immersion period, with the final rating averaging 63.23 (*SD = 20.80*).“

**Comment 3.d.:** *“Why were participants explicitly instructed to focus their attention on the test stimulus? This also seems unusual and potentially problematic as the frequent ratings of the TS (every 10 s) probably primed attention to the TS anyway. The authors state that instructions to focus on the CS can increase CPM; if this works the other way round as well, it might have led to a suppression of CPM effects.”*

**Response:** We thank the reviewer for this important comment. The rationale for explicitly instructing participants to focus on the test stimulus was to control the direction of attention across all participants. As the reviewer correctly notes, attention can influence CPM magnitude, and given that participants were required to rate the TS every 10 seconds, it is indeed likely that they were already directing their attention to it. However, to ensure uniformity and control this variable systematically, we provided explicit instructions for all participants to focus on the test stimulus.

**Comment 3.e.:** *“It is a bit unfortunate that the authors failed to obtain a classical CPM effect (main effect of condition) which might be due to the factors mentioned above. However, the time-dependent effect seems interesting (adaptation over time in both conditions, but stronger in the CPM condition) – especially in the light of previous research showing that CPM can abolish temporal summation – and could maybe be discussed a bit more”.*

**Response:** We are grateful for the reviewer’s careful evaluation of the CPM effect. In fact, there was a main effect of pain paradigm, indicating that the CPM manipulation worked as intended: pain ratings were lower during test-stimulus under conditioning compared to the test-stimulus alone. We now understand that this was not fully clear in the original manuscript, so we have revised the phrasing to make this effect explicit.

**Page 21, Paragraph 1:** “… Overall, the results indicate a main effect of pain paradigm, as pain ratings were lower for the test stimulus with conditioning compared to the test stimulus given alone (F_(1,85)_=5.89, *p*=.017, *η*^2^=.065).”

**Comment 4:** *“On p. 12, it is stated that ‘the THP test stimulus was applied for 60 seconds to the dominant hand’; I suppose the authors mean ‘**the forearm of the dominant hand’ as described in the preceding paragraph?”*

**Response:** Thank the reviewer for the clarification. This is indeed what we meant, and now we have added this to better clarify.

**Page 12, paragraph 2:** “...Then, the test stimulus was applied for 60 seconds to the forearm of the dominant hand…”

**Comment 5:** *“At some points, I was a bit confused by the terminology used for describing the experimental conditions. Personally, I prefer the terminology ‘baseline condition’ (for TS alone) and ‘CPM condition’ (for TS + CS), but I see how that would be a problem here as ‘baseline’ is used to refer to the initial HR assessment. However, I think that the term ‘THP’ could be confusing because it seems unclear if it refers to a stimulus type or a condition; this caused some confusion for me when looking at the descriptives given in Table S2. I also had to scroll back and forth a few times to understand the baseline/reactivity/recovery phases. Maybe the terminology and/or Figure 1 could be improved to simplify and clarify this.”*

**Response:** We agree that this issue warrants clarification and note the potential confusion in our terminology. As suggested, we have clarified the terminology throughout the manuscript to improve consistency and readability. Specifically, we replaced the term **“THP”** with **“test-stimulus alone”** and **“CPM”** with **“test-stimulus under conditioning stimulus”** when referring to the experimental conditions. We agree that this terminology is clearer and avoids ambiguity regarding stimulus type versus condition. In addition, we revised the corresponding text and figure descriptions (including Figure 1 and Figure 4) to ensure that the baseline, reactivity, and recovery phases are now presented in a more intuitive and streamlined manner. We believe that these revisions greatly improve the clarity of the experimental description and reduce the need for readers to refer back and forth across sections.

**Reviewer #2:**

“*This study (N=86 healthy women) tests whether attentional biases (Perceptual Load images, Dot-Probe words), psychological traits (DASS-21, PCS, FPQ), and autonomic activity (heart rate; baseline/reactivity/recovery) explain individual differences in pain sensitivity and conditioned pain modulation (CPM). Key findings: stronger interference by pain cues under high load → higher pain sensitivity; Dot-Probe avoidance → higher fear of pain; none of the examined factors predicted CPM magnitude. HR increased during CPM and stayed elevated in recovery; higher distress was related to blunted HR reactivity; attentional indices predicted higher HR during recovery.*

***Strengths***

- ***Clear gap and two-task design.*** *Nicely motivates moving beyond attentional focus to biases, using complementary tasks that target earlier capture vs strategic avoidance.*
- ***Well-specified QST/CPM and ECG stages.*** *Baseline/reactivity/recovery windows and repeated-measures analyses are clear; HR effects are quantified.*
- ***Integration of psychology and ANS.*** *Parallel treatment of distress, fear, catastrophizing with HR dynamics strengthens interpretation.*

*This manuscript represents a methodologically rigorous and theoretically well-motivated investigation into the role of attentional biases in pain sensitivity and endogenous pain modulation. The study makes meaningful contributions by demonstrating that attentional biases predict pain sensitivity independently of psychological factors, and by revealing their association with autonomic recovery dynamics, novel findings that advance our understanding of cognitive mechanisms in pain processing. The use of complementary attentional bias paradigms, careful QST/CPM methodology, and integration of autonomic measures are particular strengths. The suggested revisions focus primarily on enhancing clarity in the Discussion of unexpected null results, improving readability through strategic reorganization of methodological detail, strengthening effect size reporting, and addressing minor technical issues with figures and data accessibility.”*

**Comment 1:** *“The fact that attentional biases wasn’t correlated with CPM is unexpected. I would suggest authors to make a more scholarly discussion of this. Which could include the fact that task/modality/time differences could dilute shared variance; attentional tendencies may relate more to autonomic recovery than to CPM magnitude, which is consistent with your recovery HR findings; and most importantly that restricted range in healthy young women reduces covariance”*

**Response:** We appreciate the reviewer’s insightful comment. We have expanded our discussion to suggest why attentional biases were not associated with CPM magnitude (i.e., in Section 4.3: “Attentional biases towards pain do not affect pain inhibition efficiency”). Specifically, we discuss several important factors that may have contributed to our results, such as the distinction between implicit and explicit attentional measures and goal-directed versus automatic attentional processes. We also refer to the influence of attentional assessment during actual pain versus in the absence of pain, and link it to the broader theoretical and neural considerations differentiating early pain sensitivity from brainstem-mediated pain inhibition. These additions help clarify why attentional biases may influence pain sensitivity without necessarily predicting CPM magnitude.

Furthermore, we have added two points in the subsequent sections to fully address the reviewer’s suggestions: in Section 4.4 (“The impact of emotional distress on HR reactivity and recovery from CPM”), we highlight that attentional biases appear to affect autonomic recovery rather than CPM itself, consistent with our HR recovery findings, and in the Limitations section, we note that the restricted range in healthy young women likely reduced covariance between attentional bias and CPM measures. Together, these revisions provide a more comprehensive and mechanistic discussion of our findings.

For clarity and ease of review, we have reproduced the **entire relevant paragraph** here.
All **newly added or revised text is highlighted in blue** to facilitate tracking of the changes.

**Page 39, paragraph 1:** “The role of attention in pain modulation, particularly in CPM, remains debated [8,35]. While attention has been proposed to influence CPM [135], findings from a recent meta-analysis are mixed [8]. CPM appears to be enhanced when attention is experimentally directed toward the conditioning stimulus and reduced when attention is directed toward the test stimulus. However, studies using external distraction tasks during CPM have produced inconsistent results [8].

Unlike the goal-directed attentional processes typically examined in CPM research, attentional biases reflect more automatic, involuntary tendencies to focus on pain-related cues, typically assessed in contexts where pain is incidental rather than goal-relevant [23,25]. Our findings add a novel perspective by showing that while attentional biases are linked with increased pain sensitivity, they do not predict CPM magnitude. One potential explanation is that CPM represents a dynamic, goal-directed modulation of pain processing that is less susceptible to automatic cognitive-affective traits in healthy individuals [40]. Neural evidence may support this distinction: attentional biases engage circuits involved in threat detection and affect regulation (e.g., amygdala, ACC, orbitofrontal cortex) [18,19,28], which likely influence early pain detection and sensitivity [134]. However, CPM is dependent on brainstem-driven descending modulation, which may be less impacted by attention traits and affect regulation in healthy individuals [81,87].

A further challenge in the literature is that “attention” is often treated as a unitary construct [61,126]. Without systematic comparisons across tasks, it remains unclear whether divergent findings reflect differences in attentional stages, as proposed by Todd et al. (2015), or instead arise from variation in task characteristics. One important distinction concerns whether attentional capture is assessed implicitly (e.g., reaction-time interference paradigms) or explicitly (e.g., self-reports of noticing or attending to specific stimuli) [117]. These approaches do not necessarily index the same attentional mechanisms and may be differentially influenced by cognitive control processes [24,117].

This distinction is particularly relevant when comparing our findings with recent work on intrinsic attention to pain, which reflects a more explicit, trait-like attentional measure [3]. Adams et al. (2021) assessed pain-related attentional tendencies by asking participants to report the extent to which their thoughts were focused on pain during brief noxious stimulation. They found that higher self-reported intrinsic attention to pain predicted both less efficient pain inhibition and greater pain amplification [3]. In contrast to this explicit approach, our tasks assessed attention to pain implicitly, with participants unaware of the relevance or meaning of the pain-related cues.

Notably, the dual-pain CPM paradigm requires participants to deliberately attend to ongoing pain in order to provide continuous verbal ratings. Therefore, explicit attentional tendencies may be more relevant and more directly linked to CPM efficiency measured by self-report than implicit attentional biases. This pattern is consistent with findings from other domains, where implicit and explicit measures influence behavior differently [14,50]. For example, attentional biases toward exercise cues are associated with positive implicit attitudes (i.e., automatic mental associations between an object and its evaluation) toward physical activity. However, only explicit attitudes toward physical activity (i.e., self-reported opinions and intentions) moderate whether these attentional biases translate into actual sport behavior [14]. Collectively, these observations raise the hypothesis that explicit attention toward pain may moderate the relationship between attentional biases and CPM efficiency, a possibility that warrants investigation in future studies.

A second difference between our study and Adams et al. (2021) concerns whether attentional biases are assessed while participants are actually experiencing pain. Pain-related cues presented in the absence of pain may be insufficient to capture the complexity of attentional tendencies to pain in healthy individuals, as they do not evoke the motivational significance, emotional arousal, or regulatory demands that characterize real pain [105,106,123]. Assessing attentional biases during ongoing nociceptive input may therefore provide a more sensitive and ecologically valid indication of how individuals naturally prioritize and process pain-relevant information. This point is particularly important given the scarcity of studies that have examined attentional biases in the context of actual pain, despite evidence that such biases are meaningfully related to pain cognitions and behaviors [6,66,104]. For instance, Van Ryckeghem et al. (2013) demonstrated that in chronic pain populations, attentional biases interact with daily pain severity to shape disability and distractibility, illustrating that attentional patterns during pain can have meaningful behavioral consequences [104]. This underscores the relevance of further investigating attentional biases under conditions of real nociception to better understand how they manifest and operate in everyday pain experiences [125].

Taken together, our findings emphasize that attention is a multifaceted construct in pain research. Differences between attentional types—such as strategic versus automatic, internal versus external, and explicit versus implicit—likely contribute to inconsistent findings linking attention, pain sensitivity, and pain inhibition. Future studies should measure both implicit and explicit attention, ideally within the same experimental design and in the presence and absence of pain, to clarify how these processes individually and jointly influence endogenous pain modulation in clinical and non-clinical populations.”

**Page 42, paragraph 2**: “… This pattern may help explain why attentional biases were not associated with CPM magnitude, as their influence appears to emerge primarily during recovery-related processes where affective–physiological regulation plays a major role, instead of during endogenous pain inhibition. ...”

**Page 43, paragraph 1:** “…In addition, the relative homogeneity and low psychological distress typically observed in healthy young samples likely restricted variability in both attentional bias indices and CPM responses, reducing the covariance needed to detect associations between them. …”

**Comment 2:** “*The authors have done an exemplary job providing comprehensive methodological details, which greatly enhances transparency and reproducibility. However, the level of detail in the main text occasionally impedes narrative flow and may overwhelm readers unfamiliar with attentional bias paradigms or QST protocols. I recommend moving some procedural specifics to supplementary materials while retaining core conceptual information in the main text. For example, Section 2.3.1.a (Perceptual Load task, lines 156-182) could be streamlined to emphasize what the task measures (early attentional capture by pain stimuli under varying cognitive load) and why it was chosen, with stimulus presentation parameters, letter selection criteria, and catch trial percentages moved to a supplement. The same could be done for other procedures. This restructuring would help readers grasp the conceptual logic—how attentional biases were operationalized and measured—without getting lost in technical specifications, while still providing the detail necessary for replication”*

**Response:** We thank the reviewer for this very helpful suggestion. As recommended, we streamlined Section 2.3 (Perceptual Load and Dot-Probe Task) to improve narrative flow and to emphasize the conceptual purpose of the task rather than technical details. In the revised manuscript, the main text now focuses on: What the tasks measure, the underlying logic of the paradigms, and how the biasindexes were computed.

To enhance readability, we moved several procedural details to the Supplementary Materials (Section 1). These include timing parameters, spatial layout of stimuli, word-pair and images selection, counterbalancing procedures, practice-trial structure, and the full stimulus list. The streamlined main-text description now provides the conceptual rationale and behavioral outcome measures, while all methodological specifics necessary for replication remain fully accessible in the supplement. We believe this restructuring improves clarity for readers unfamiliar with attentional bias paradigms, while maintaining methodological transparency, as you suggested.

### Page 9: “2.3.1. Attentional bias tasks.

For ease of reading, attentional bias task details are presented here briefly. All procedural details, including stimulus timing, word presentation locations, counterbalancing scheme, word-set construction, probe identities, practice trial structure, and the complete list of stimuli, are provided in **Supplementary Materials (Section 1)** to ensure full transparency and reproducibility.

2.3.1.a. Modified Perceptual Load task. Attentional capture by pain-related information under differing cognitive demands was assessed using a modified version of the Perceptual Load paradigm [59], adapted from our previous work [87,89]. In this task, participants identified a target letter (X or N) presented under either **low** or **high perceptual load**, operationalized by the number of non-target letters surrounding the target. High-load trials required greater attentional resources, thereby reducing available capacity for processing irrelevant information.

On each trial, a pain-related or neutral image briefly appeared at fixation while participants performed the letter-identification task. Participants were instructed to ignore the images and respond as quickly and accurately as possible. Attentional interference was quantified as a **pain-related interference index**, calculated as the difference in reaction times (RTs) between pain-image and neutral-image trials, separately for low- and high-load conditions (see Fig 2).

2.3.1.b. Dot-Probe task. Attentional orienting toward pain-related information was further assessed using a modified Dot-Probe task commonly applied in pain research [108,114]. The task measures attention bias by simultaneously displaying one pain word and one neutral word on the screen, after which one of them is replaced by a letter probe. In this task, although participants were not required to respond to pain or neutral stimuli directly, attentional capture is inferred from RT to the probe appearing in a location previously occupied by either a pain or a neutral word.

Participants were asked to identify the probe letter as quickly and accurately as possible, and an **attention bias score** was computed by subtracting the mean reaction time of pain-congruent trials (probe replacing the pain word) from pain-incongruent trials (probe replacing the neutral word).”

**Comment 3:** *“While the authors report η² for their ANOVA analyses, I recommend also including Cohen's d effect sizes for key pairwise comparisons and t-tests to facilitate meta-analytic integration and practical interpretation. It is recommended to report both η² for omnibus tests and Cohen's d (or Hedges' g for small samples) for specific contrasts, as this provides readers with both variance explained and the magnitude of differences in standard deviation units. This is particularly important given that some findings (e.g., attentional biases predicting pain sensitivity) represent novel contributions that future researchers will want to interpret and replicate.”*

**Response:** We welcome this comment and fully agree with the recommendation to provide standardized effect sizes that support future meta-analytic integration and enhance the interpretability of our results. In light of this, and given the importance of facilitating replication of our findings, we have now added **Hedges’ g** for all relevant pairwise comparisons in the reported tables and t-tests throughout the manuscript, see for example:

**Page 19, Paragraph 1:** “There was a significant difference in accuracy between the load conditions of the Perceptual Load task, with higher accuracy in the low load condition (Mean accuracy: 0.94) than in the high load condition (Mean accuracy: 0.83; t_(85)_=12.4, *p*<.001, **Hedges’ g = 1.34**; see Table S2)…..

…. interference from pain-related images emerged under low load (t_(85)_=3.074, *p*=.003, **Hedges’ g = 0.316**)…”

**Page 20, Paragraph 2:** “Pain ratings collected after the first 20 seconds of hand immersion averaged 44.55 (*SD = 16.57*) and significantly increased over the 2.5-minute immersion period, with the final rating averaging 63.23 (*SD = 20.80;* t_(83)_= 8.56*, p<.001,* **Hedges’ g = 0.936**).”

**Comment 4:** *“The sentence “Specifically, the ANS is involved in shaping CPM efficacy, with studies showing increased 96 heart rate (HR) and sympathetic activation during CPM: should be revised to distinguish group-level HR increases “during CPM” from between-person prediction. Do authors mean that the expected within-task HR rise or that individual HR reactivity predicts CPM magnitude”*

**Response:** We thank the reviewer for this valuable clarification request. We revised the paragraph to clearly distinguish between-person predictors of CPM from within-task cardiovascular changes during CPM. Specifically, we now state that resting vagal tone predicts later CPM magnitude and that greater sympathetic reactivity during pain is associated with enhanced CPM efficacy—both reflecting between-person associations. We also explicitly note that task-evoked autonomic changes during CPM (i.e., group-level decreases in vagal tone and increases in sympathetic activation) have been examined in only two studies and, in the one study that tested it, did not correlate with CPM magnitude.

**Page 5, Paragraph 3:** “In addition, autonomic nervous system (ANS) activity is increasingly recognized as a key contributor to the subjective experience of pain [19,99] and pain inhibition [13,53,63]. Specifically, initial evidence suggests that the ANS may be involved in shaping CPM efficacy. A few studies have linked resting vagal tone, measured via heart rate variability (HRV) or blood pressure, to later CPM magnitude, with higher vagal tone predicting a larger CPM effect [69,72,76]. Other work highlights the role of sympathetic activation, showing that greater sympathetic reactivity during pain alone is associated with enhanced CPM efficacy [15,119]. At the same time, cardiovascular autonomic dynamics during CPM have been investigated in only two studies to date [5,51]. Both reported group-level decreases in vagal tone and increases in sympathetic activation during the CPM procedure; however, only one study examined whether such changes were associated with CPM magnitude, and it found no significant correlation [51]. “

**Comment 5:** *“At first mention in Participants, add a parenthetical brief explanation regarding SONA, clarifying its meaning. This makes recruitment transparent for international readers and non-academic audiences.”*

**Response:** We agree that this issue warrants clarification, as the original text was not fully clear. To address this, we added a brief parenthetical clarification at the first mention of SONA in the Participants section to explain its meaning. This ensures that recruitment procedures are transparent for international readers and non-academic audiences.

**Page 7, Paragraph 1:** “Participants were recruited through the university's research SONA participation system (an online system used to manage and screen student research participation) ...”

**Comment 6:***“Authors describe that normality was checked using Shapiro-Wilk and histograms but don’t describe what was done if violated.”*

**Response:** We thank the reviewer for this comment and have clarified this issue in the manuscript. Information regarding normality checks had previously been reported in multiple sections of the Methods (e.g., Section 2.4.1 for reaction times in the attentional tasks and Section 2.4.2 for heart rate measures), but we recognize that this presentation was not sufficiently clear or well organized. We now explicitly state at the beginning of the Data Pre-processing section that the only variable deviating from normality was the DASS score, which showed a left-skewed distribution. This pattern is expected in a healthy, non-clinical sample, as emotional distress measures often show non-normal distributions in the general population. Given the sample size (>50) and the robustness of linear regression to mild deviations from normality in predictor variables, no transformation was applied and the original DASS values were retained. This decision is now clearly stated in the revised manuscript.

**Page 14, Paragraph 3:** “Normality was assessed for all variables using visual inspection via histogram plots and the Shapiro-Wilk normality test. The DASS scores showed left-skewed distribution, a pattern expected in a healthy, non-clinical sample, as emotional distress measures often display skewed distributions in the general population (see, for example, [19,104]). Given the sample size (>50) and the robustness of linear regression to mild non-normality in predictor variables, no transformations were applied, and the original DASS values were retained.”

**Comment 7:** *“The resolution (DPI) of the figures appears to be below publication standards. Panel labels in Figure 6 (A, B, C) are difficult to read, and the violin plot contours show pixelation.”*

**Response:** We thank the reviewer for pointing this out. We have replaced all figures with higher-resolution versions (≥300 DPI) and ensured that panel labels and plot details are now clearly legible, including improved clarity of the violin plot contours in Figure 5.

**Comment 8:** “*The OSF data repository link provided (*[*https://osf.io/3bwy7/files/osfstorage*](https://osf.io/3bwy7/files/osfstorage)*) does not appear to be functioning properly or may not be publicly accessible. When accessing the link, it does not resolve to a viewable repository page. I believe the correct link should be:* [*https://osf.io/3bwy7/files*](https://osf.io/3bwy7/files) *or* [*https://osf.io/3bwy7*](https://osf.io/3bwy7).”

**Response:** We thank the reviewer for noticing this issue. We have corrected the OSF link, and the data are now publicly accessible at: [OSF | The involvement of attentional biases in endogenous pain inhibition](https://osf.io/3bwy7/overview). All files associated with the study have been uploaded and are fully accessible through this repository.

**Reviewer #3:**

*“This study investigated whether attentional bias predicts individual differences in pain sensitivity and endogenous pain modulation, and whether psychological, emotional, and autonomic factors provide additional explanatory value. The authors are to be congratulated for this work. The methods in particular were very clearly described. Well done!
I have only a few minor queries:”*

**Comment 1:** “*The quality of the figures seems to be low; they are quite pixelated. I recommend that you increase the resolution of the images*.”

**Response:** We thank the reviewer for this helpful and important comment. We have replaced all figures with higher-resolution versions (≥300 DPI) and ensured that panel labels and plot details are now clearly legible.

**Comment 2:** “ *Section 2.4.1: please provide a justification for including only participants with 2.5 z-scores above or below each participant’s average RT. The rationale for this approach is not entirely clear.”*

**Response:** We have addressed this point as follows: Participants whose average RTs deviated more than ±2.5 standard deviations from the group mean were excluded to remove cases with overall atypical performance, which could indicate inattention, misunderstanding of the task, or other factors that make their data non-representative. This is a standard approach in cognitive research to ensure that analyses reflect typical task-focused performance. In our sample, applying this criterion resulted in the exclusion of only one participant from the Perceptual Load task. Based on the reviewer's recommendation, the rationale is now clearly stated in the manuscript.

**Page 15, Paragraph 2:** “Participants whose **average reaction times deviated more than ±2.5 standard deviations from the group mean** were excluded from analysis, in order to remove cases with atypical overall performance that could reflect inattention, misunderstanding of the task, or other non-representative behavior. Using this criterion, data from one participant were excluded from the Perceptual Load task.”

**Comment 3:** “*The relevance of including BMI and ethnicity are unclear. Please can you include a justification for the relevance of these outcomes.”*

**Response:** We thank the reviewer for this clarifying comment. BMI was assessed because it can influence heart rate, allowing us to check for potential confounding effects. In our data, BMI was not associated with heart rate at rest or during pain and thus was not included as a covariate. Ethnicity (Arab vs. Jewish) was recorded as another characteristic of our sample, and to examine whether mother tongue influenced performance on the Dot-Probe task, which included reading words in Hebrew. We found no significant effects of ethnicity on task performances. These clarifications have been added to the Methods and the results section, as indicated below:

**Page 8, Paragraph 2:** “…. completed the baseline assessment, including demographics (e.g., age, Body mass index (BMI), ethnicity), psychological traits, and emotional state (PCS, FPQ-9, and DASS-21; see Fig 1). BMI was recorded because it can influence HR, allowing us to evaluate potential confounding effects on autonomic measures. Participants then completed two attentional bias tasks (Perceptual Load and Dot-probe), with instructions provided both on-screen and verbally. At the beginning of each task, practice trials were used to ensure task comprehension and were repeated once if performance accuracy was low. Ethnicity (Arab vs. Jewish) was recorded to describe the sample and to assess whether mother tongue influenced performance on the Dot-Probe task, which contained Hebrew words.

**Page 18, Paragraph 3:** “In this sample, BMI was not associated with HR at rest or during pain, and ethnicity did not significantly affect task performance (all *ps*>.15). These variables were therefore not included as covariates in subsequent analyses.”

**Comment 4:** *“In Table 1, it would be useful for the reader if you include the possible ranges of the questionnaires so that the reader can better interpret your summary statistics.”*

**Response:** We appreciate the reviewer’s feedback regarding Table 1 and agree that including the possible score ranges improves the interpretability of the summary statistics. We have now added the possible ranges for all questionnaires to Table 1.

**Comment 5:** *“Please can you include assessments of model fitness for your regression analyses”.*

**Response:** This is an important point, and we appreciate the reviewer for raising it. In response, we have clarified and expanded the assessment of model fit in all regression analyses. Specifically, we now report the standard error of the estimate (SEE), R², Adjusted R², ΔR², the corresponding F-change statistics, and the Durbin–Watson statistic to evaluate independence of residuals. These indices provide a comprehensive overview of overall model fit, predictive accuracy, and assumption compliance. We have added these values in the Results section and incorporated them into the corresponding regression tables to ensure transparency and interpretability of the model performance.

**See, for example, Page 24, paragraph 3 (changes are highlighted in blue for clarity):** “The results of the block hierarchical multiple regression predicting pain sensitivity, as measured by pain-60 temperature, are presented in Table 4. **Block 1,** which included attentional bias measures, explained 10.4% of the variance in pain-60 temperature, with a standard error of estimate of 1.89 [F_(3,76)_=2.94, *p*=.035, R²= .104, Adjusted R²=0.07, SEE= 1.89]. Specifically, higher pain interference under both low (β=-0.19, 95% BCa CI [-0.025, -0.002], *p*=.020) and high perceptual loads (β=-0.25, 95% BCa CI [-0.017, -0.002], *p*=.015) predicted lower temperatures rated as pain-60, indicating greater pain sensitivity. Collinearity diagnostics indicated no multicollinearity concerns (VIFs < 1.2), the residuals were approximately normally distributed and homoscedastic, with no autocorrelation (Durbin–Watson = 2.20). Standardized residuals were within ±3 SD, indicating no extreme outliers.”

**Comment 6:** “*Was the protocol/statistical analysis plan locked online prior to conducting the analysis? In acknowledgement of the principles of open science, I'd recommend reporting on this”*

**Response:** The study protocol and statistical analysis plan were not preregistered online prior to data analysis. Although the study was guided by a priori, theory-driven hypotheses regarding the involvement of attentional bias, psychological distress, and autonomic activity in endogenous pain modulation, it was exploratory with respect to the relative contribution and combined explanatory value of these factors. All analyses are reported transparently, and results are interpreted cautiously.

We thank the reviewers and editorial team once again for their careful reading and helpful suggestions. We believe the manuscript is significantly improved as a result, and we hope it is now suitable for publication in *Plos One*.

Sincerely,
Einav Gozansky (on behalf of all authors)
